# Regulation Strategies, Contextual Problems, Addictive and Suicidal Behaviors: A Network Perspective with Adolescents

**DOI:** 10.3390/bs14121236

**Published:** 2024-12-23

**Authors:** Dalila Eslava, Begoña Delgado, Miguel Á. Carrasco, Francisco Pablo Holgado-Tello

**Affiliations:** 1 Faculty of Health Sciences, Valencian International University, 46002 Valencia, Spain; dalila.eslava@universidadviu.com; 2 Department of Developmental and Educational Psychology, Faculty of Psychology, National University of Distance Education, 28040 Madrid, Spain; bdelgado@psi.uned.es; 3 Department of Personality, Assessment and Psychological Treatment, Faculty of Psychology, National University of Distance Education, 28040 Madrid, Spain; macarrasco@psi.uned.es; 4 Department of Methodology of Behavioural Sciences, Faculty of Psychology, National University of Distance Education, 28040 Madrid, Spain

**Keywords:** adolescence, emotional regulation, addictions, suicidal behavior, psychometric networks

## Abstract

Adolescence is a period marked by challenges, including problems that appear in the adolescent’s context. To manage these, adolescents use a series of emotional regulation skills that can be more or less adaptive. Less adaptive regulation is related to problem behaviors such as alcohol abuse, substance addiction, problematic internet use, and/or suicidal behavior. This study employs psychometric networks to analyze the association between these problem behaviors, the existence of contextual problems, and the use of cognitive emotional regulation strategies. We performed this analysis for the total sample: the male sample and the female sample. The total sample consists of 758 participants; 424 females (55.4%) and 341 males (44.6%) between the ages of 12 and 21 years (*M* age = 15.85; *SD* = 2311). The results show that less adaptive regulation strategies are the most central node, exhibiting a positive relationship with problem behaviors and contextual problems. In contrast, adaptive regulation strategies are a less influential node. Finally, problem behaviors are related to each other. Differences emerged between the male sample and the female sample. These findings contribute to improving our understanding of the phenomenon as well as to the construction of preventive interventions.

## 1. Introduction

Adolescence is a life stage of great strength and potential that extends approximately from the age of 12 to 18, or even 21 [1]. This evolutionary stage is characterized by the need to face, within a relatively short time, various physical, cognitive, psychological, and social challenges and changes [2] that can reveal vulnerabilities and, at times, present difficulties [3]. Appropriate adaptation to these challenges and changes can facilitate the transition to adulthood [2]. However, for some young people these changes can lead to psychosocial maladjustment [4,5,6,7,8] that can have a significant impact on adulthood [9].

Substance use often appears for the first time during adolescence, and some authors point out that, for many adolescents, it can represent a ritual of transition between adolescence and adulthood [10,11]. According to data from the ESTUDES survey (State Survey on Drug Use in Secondary Education) [12], when consulted about their consumption of certain substances within the previous 30 days, 56.6% of Spanish adolescents reported having used alcohol, 21% having used tobacco, and 15.6% having used cannabis. Contact with these substances in adolescence is a concern, not only because of the neurocognitive consequences [13,14] but also because consumption at younger ages often leads to higher rates of consumption in adulthood [15]. Likewise, adolescence is a vulnerable period for problematic internet use [16], which has become a public health concern [17]. Asked about their internet use in the previous 30 days, 23.5% of Spanish adolescents revealed compulsive internet use [18], which can lead to psychological, social, and/or academic difficulties [19].

On the other hand, adolescence is also a vulnerable period in terms of emotional problems, such as suicidal behavior [20]. At this stage, these types of thoughts appear for the first time, along with the first existential crises [21]. The PSICE (Evidence-Based Psychology in Educational Contexts) report estimates that 5.4% of adolescents experience suicidal ideation and that 4.9% have attempted suicide [7]. In fact, suicide is the leading cause of unnatural death among adolescents between 15 and 19 years of age in Spain [22]. Suicidal behavior can be manifested in a variety of ways, from suicidal ideation and planning, through suicidal communication, to suicide attempts and death by suicide [11,23].

These behavioral and emotional problems are interrelated. For example, the prevalence of heavy alcohol use, daily tobacco use, and cannabis use is higher in those adolescents who are prone to compulsive internet use [18]. In addition, substance use is related to suicidal behavior [24], as is problematic internet use [25,26].

These problem behaviors share triggers, risk factors, and maintenance factors [27], one of which is difficulty in emotional regulation [28,29]. Garnefski and Kraaij [30] state that cognitive emotional regulation is the process by which people influence how they experience and express their emotions, and it plays an important role in emotional and behavioral problems. At this stage, adolescents may have to manage demands and challenges [2], and may face problems in their family, social, and/or school contexts [4]. In this sense, it is important to consider the conditions presented by the contexts in which an adolescent develops, especially when such conditions are problematic. To cope with these new challenges, adolescents can count on adaptive regulation skills that allow them to manage their emotions appropriately. However, when these skills are not available, the adolescent may embark on a series of ruminative thoughts, giving rise to intense emotions, as well as less adaptive behaviors to cope with them [31,32]. For example, substance use can be a way to cope with stressful situations [33]. Likewise, suicidal thoughts seem to function as a form of emotional regulation for some individuals [34]. Therefore, assuming that contextual problems are crucial to understanding an adolescent’s problem behaviors, the adolescent’s emotional regulation plays a key role in determining their impact [35].

It is important to consider gender differences when examining these phenomena. Regarding the prevalence of the aforementioned behaviors, boys have higher rates of cannabis use [12] and compulsive internet use [18], while girls have higher rates of alcohol and tobacco use [12]. Likewise, according to the PSICE study, girls provide a higher proportion of affirmative responses than boys when suicidal behavior is evaluated [7]. However, Espada-Sánchez et al. [25] find that there is a “gender paradox” by which women attempt suicide more frequently, but death by suicide occurs to a greater extent in men. This is partly attributed to the higher prevalence of affective disorders (e.g., depression and anxiety) in girls and the tendency of boys to use more lethal methods of suicide such as hanging or firearms [36]. On the other hand, several authors indicate that boys are more likely than girls to use substances as an emotional regulation strategy [37,38]. However, it seems that girls have greater emotional difficulties [7]. These differences may be due to socially established gender roles, whereby adolescents respond differently to the demands of the adolescent life stage and the problems that arise during it [39]. Therefore, it is important to identify and address these differences when studying these phenomena.

The main aim of this study is to explore the association between problem behaviors (alcohol abuse, substance addiction, problematic internet use, and suicidal behavior), the existence of contextual problems (as a general measure, including indistinctly problems related to the family, school, or relationships with peers), and the use of cognitive emotional regulation strategies (adaptive regulation and less adaptive regulation). This approach is also carried out according to gender. To our knowledge, the existing literature has addressed these relationships in isolation [32,40]. Less adaptive regulation is expected to be positively related to problem behaviors, while more adaptive regulation is expected to be negatively related to problem behaviors. Regarding contextual problems, it is expected that they have a positive relationship with less adaptive regulation strategies, as well as with problem behaviors. Problem behaviors are also expected to have a positive relationship with each other. Regarding the difference between boys and girls, taking into account the differences presented by the literature, it is expected that substance addiction and problematic internet use are more intensely related to emotional regulation strategies in boys, while in girls these relationships pivot on alcohol and tobacco abuse and suicidal behavior. However, this study goes beyond establishing specific hypotheses according to the literature by employing an exploratory perspective based on psychometric networks. Thus, an essential and key objective is to identify which variables are most relevant in the proposed network and how they interact with each other, with the goal of better understanding this complex phenomenon.

## 2. Materials and Methods

### 2.1. Participants

The final sample of the study comprised 765 participants, 424 female (55.4%) and 341 male (44.6%), all of them between the ages of 12 and 21 years (*M* age = 15.85; *SD* = 2311). The data were collected in 2023, between January and November. Most of them were high school students (95%), and only 37 participants were university students. The sample was selected from various secondary schools and colleges using non-random and convenience sampling. Given this sampling design, only two regions of Spain were represented: Madrid (79.81%), mainly, and Andalusia.

### 2.2. Instruments

- Sociodemographic data. An ad hoc questionnaire was administered to collect sociodemographic information (age and sex).
- MULTICAGE CAD-4 [41]. This instrument assesses addictive disorders through eight subscales. For the present study, only the subscales of alcohol abuse/dependence, substance addiction, and internet addiction were used. Respondents answered using “yes” or “no”. The subscales have a Cronbach’s alpha index above 0.7 [41].
- SENTIA-brief [42]. The Adolescent Suicidal Behavior Assessment Scale was used, specifically the brief 5-item modality. This scale consists of a general factor, suicidal behavior, and three subscales: ideation, communication, and performance/planning. For the present study, the global score was used. The answers follow a five-point Likert scale (1 = “never” and 5 = “many times”). The omega coefficient for the total score is 0.97 [42].
- Cognitive Emotion Regulation Questionnaire (CERQ-short) [43,44]. This is a reduced version of the 18-item CERQ questionnaire. It is composed of two subscales: adaptive regulation (evaluating strategies such as replanning, reevaluating, accepting, and focusing on the positive) and less adaptive regulation (evaluating catastrophizing, rumination, blaming oneself, and blaming others). The answers follow a five-point Likert scale (1 = “almost never” and 5 = “almost always”). In the Spanish version, the Cronbach’s alphas of the scales are 0.84 and 0.72, respectively [44].
- Child and Adolescent Assessment System (SENA). This is a global index of contextual problems [45]. It is an instrument for detecting a wide spectrum of emotional and behavioral problems in children and adolescents, as well as areas of vulnerability and psychological resources. The present study uses the “contextual problem index”, assessing family-, school- and peer-related aspects. The answers follow a five-point Likert scale (1 = “never or almost never” and 5 = “always or almost always”). A high score on this index is an indicator of a greater severity of mismatch across different contexts and not exclusively in one of them. Direct scores are used.

### 2.3. Procedure

The study is part of a broader project on adolescents’ behavioral problems. It was approved by the ethics committee of the Universidad Nacional de Educacion a Distancia in compliance with the ethical standards established in Law 3/2018 on Data Protection and Guarantee of Digital Rights. Accordingly, it required informed consent to be obtained from both the adolescents and their parents, and participation was voluntary. All data were kept anonymous and confidential. After sampling, data were collected by previously trained researchers in small groups during a 90 min session.

### 2.4. Data Analysis

The study first conducted a descriptive analysis to obtain the frequencies and other statistics of the problem behaviors for the total sample, as well as for the boys and girls separately. To gain a preliminary understanding of the relationship between the variables, bivariate correlations were performed on the three samples. Two analyses were performed to identify differences between the sample of boys and the sample of girls: the Kolmogorv-Smirnov normality test, followed by the non-parametric Mann-Whitney U test. The SPSS v27 statistical package was used.

To delve deeper into the relationship between the variables, a psychometric network analysis was conducted on the total sample, as well as separately for boys and girls, to explore the relationships between the variables. This analysis graphically represents a model that collects statistically significant relationships; it is composed of nodes (study variables) and edges (relationships between the nodes). In this case, the network was weighted undirected and the pcor method was used, with a *p* < 0.05 significance for the edges. The qgraph and bootnet libraries of R were used. Four measures of centrality were estimated: strength (St), closeness (Clo), intermediation (Bet), and expected influence (EI). The strength of a node refers to the sum of the absolute partial correlation coefficients between one node and all other nodes, i.e., a node with a high strength is a node that influences many other nodes. Proximity refers to how strongly a node is directly related to the rest of the nodes, i.e., a node with high proximity is a node that can predict other nodes well. Intermediation indicates how many of the shortest paths between two nodes pass through the node in question, so if a node has a high brokerage value, this indicates that it is well connected to the rest of the nodes in the network. Finally, expected influence uses the sum of the absolute weights of all the edges of a node, i.e., converting negative edges into positive edges before adding. For this reason, it is presented as a more precise index of centrality [46,47]. However, Bringmann et al. [48] indicate that for the study of psychological variables, proximity and intermediation indices are inadequate to establish the importance of nodes in the network. Their values are unstable in cross-sectional studies, showing wide confidence intervals and, in some cases, incoherence.

Likewise, precision and stability measurements were obtained. The accuracy chart shows the estimated edge value in the sample (red line) and the 95% confidence intervals (gray bars) for those estimates. Finally, the stability graph assesses the consistency of the estimators through resampling procedures, replicating the network with fewer cases. The lines indicate the correlation between the estimates of the indices in the full sample and of those indices when the sample is reduced. If the correlation falls below 0.70, the estimates become unstable, and it is recommended that it should not fall below 0.70 with half of the sample [47,49].

## 3. Results

We tested whether there were differences between boys and girls on the measured variables. To determine which statistic to use, we first performed the Kolmogorov-Smirnov normality test; as the variables did not follow a normal distribution, we next performed the non-parametric Mann-Whitney U-test and compared the mean ranks between the samples of boys and girls. The results show that there are statistically significant differences between some of the variables. Girls exhibited more problematic internet use (*p* < 0.05) as well as more suicidal behavior (*p* < 0.001) and less adaptive regulation (*p* < 0.001) than boys (means and standard deviations are included in Table 1).

Table 2 shows the bivariate correlations of the three samples.

### 3.1. Psychometric Network Analysis of the Total Sample

**Figure 1:**
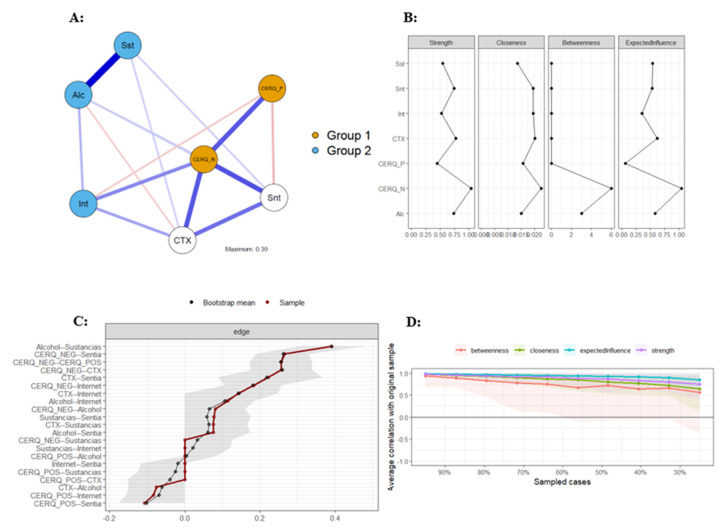
Network analysis of regulation strategies, contextual problems, and addictive and suicidal behaviors in the total sample. Alc/Alcohol = alcohol abuse; Sst/Sustancias = substance addiction; Int/Internet = problematic internet use; Snt/Sentia = suicidal behavior; CTX = contextual problems; CERQ_P/CERQ_POS = adaptative behavior; and CERQ_N/CERQ_NEG = less adaptative behavior. Panel (**A**): Estimated network graph in total sample; Panel (**B**): Non-standardized estimates of the centrality measures in total sample; Panel (**C**): Precision plot of estimated network in total sample; Panel (**D**): Stability indices of the edges in total sample.

Figure 1 shows the results of a psychometric network analysis of the total sample. Panel A shows the estimated network of regulation strategies, contextual problems, and addictive and suicidal behaviors. The network is interconnected. Panel B of Figure 1 presents non-standardized estimates of the centrality measures; according to Isvoranu et al. [50], these are the values that should be reported. The most central node in terms of strength, expected influence, and betweenness is less adaptive regulation strategies (St = 1.04; EI = 1.04; Bet = 6; and Clo = 0.02). Another variable that stands out is the presence of contextual problems (St = 0.77; EI = 0.62; Bet = 0; and Clo = 0.20). The results for the other variables are as follows: suicidal behavior (St = 0.75; EI = 0.53; Bet = 0; and Clo = 0.02), alcohol abuse (St = 0.74; EI = 0.58; Bet = 3; and Clo = 0.02), substance addiction (St = 0.54; EI = 0.54; Bet = 0; and Clo = 0.01), problematic internet use (St = 0.52; EI = 0.36; Bet = 0; and Clo = 0.02), and adaptive regulation (St = 0.45; EI = 0.07; Bet = 0; and Clo = 0.02).

In panel C of Figure 1, the precision plot shows that the estimated network demonstrated adequate precision. In most cases, the estimates obtained through resampling (n = 1000) overlap with the original estimates, and none fall outside the confidence interval. Panel D of Figure 1 presents the stability indices of the edges in the estimated network. When the sample size is reduced by 50%, the estimates do not fall below 0.70. These results indicate that the estimated network is stable, and the conclusions drawn from it can be generalized.

### 3.2. Psychometric Network Analysis in the Boys’ Sample

**Figure 2:**
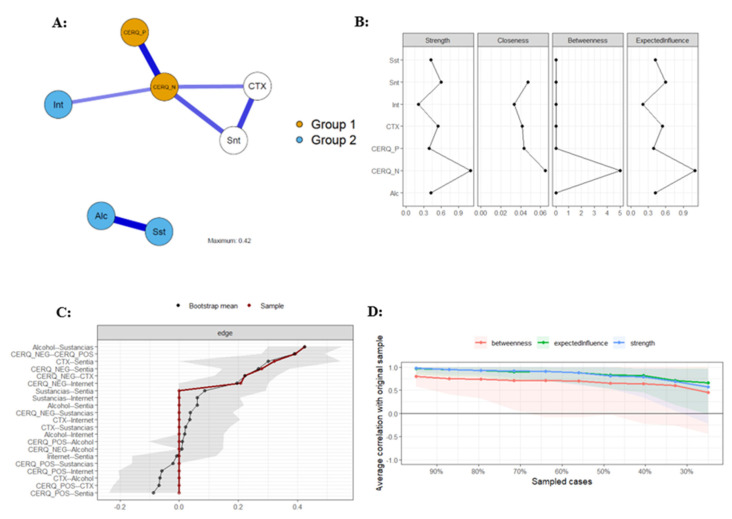
Network analysis of regulation strategies, contextual problems, and addictive and suicidal behaviors in the male sample. Alc/Alcohol = alcohol abuse; Sst/Sustancias = substance addiction; Int/Internet = problematic internet use; Snt/Sentia = suicidal behavior; CTX = contextual problems; CERQ_P/CERQ_POS = adaptative behavior; and CERQ_N/CERQ_NEG = less adaptative behavior. Panel (**A**): Estimated network graph in boys; Panel (**B**): Non-standardized estimates of the centrality measures in boys; Panel (**C**): Precision plot of estimated network in boys; Panel (**D**): Stability indices of the edges in boys.

Figure 2 presents the psychometric network analysis for the male sample. Panel A shows the estimated network of regulation strategies, contextual problems, and addictive and suicidal behaviors. The network is interconnected. Panel B of Figure 2 presents the non-standardized estimates of the centrality measures. The most central node in terms of strength, standardized influence, and betweenness is less adaptive regulation strategies (St = 1.10; Clo = 0.06; Bet = 5; and EI = 1.10). Another notable variable is suicidal behavior (St = 0.60; Clo = 0.05; Bet = 0; and EI = 0.60). The results for the other variables are as follows: contextual problems (St = 0.54; Clo = 0.04; Bet = 0; and EI = 0.54), alcohol abuse (St = 0.42; Clo = 0.04; Bet = 0; and EI = 0.42), substance addiction (St = 0.42; Clo = NA; Bet = 0; and EI = 0.42), adaptive regulation strategies (St = 0.39; Clo = 0.04; Bet = 0; and EI = 0.39), and problematic internet use (St = 0.21; Clo = 0.03; Bet = 0; and EI = 0.21).

In panel C of Figure 2, the precision plot indicates that the estimated network does not demonstrate adequate precision in the estimation. There are discrepancies between the sample and the resampling (n = 1000) as most nodes show estimates different from zero, which alters the interpretation of the network. Panel D of Figure 2 presents the stability indices of the edges in the estimated network. In this analysis, the stability of the strength and influence of the variables does not fall below 0.70 when the sample is reduced by 50%, except for strength. The estimation of closeness shows no variance, so the statistical program does not display it. These results suggest that the estimated network is partially stable; therefore, caution should be used when generalizing its results.

### 3.3. Psychometric Network Analysis in the Girls’ Sample

**Figure 3:**
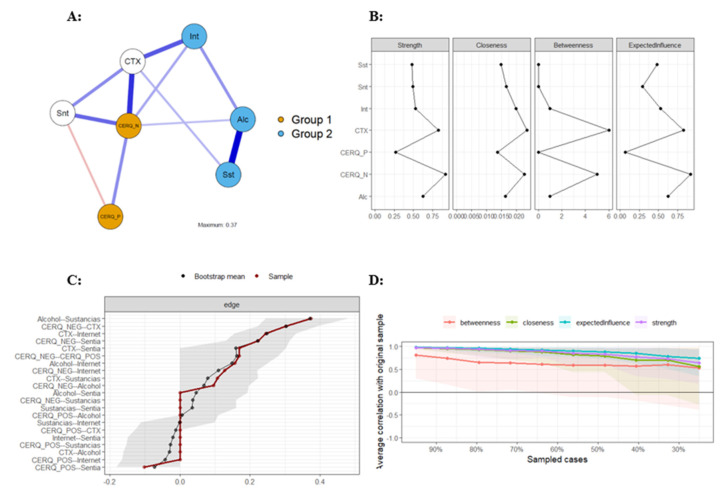
Network analysis of regulation strategies, contextual problems, and addictive and suicidal behaviors in the female sample. Alc/Alcohol = alcohol abuse; Sst/Sustancias = substance addiction; Int/Internet = problematic internet use; Snt/Sentia = suicidal behavior; CTX = contextual problems; CERQ_P/CERQ_POS = adaptative behavior; and CERQ_N/CERQ_NEG = less adaptative behavior. Panel (**A**): Estimated network graph in girls; Panel (**B**): Non-standardized estimates of the centrality measures in girls; Panel (**C**): Precision plot of estimated network in girls; Panel (**D**): Stability indices of the edges in girls.

Figure 3 presents the psychometric network analysis for the female sample. Panel A shows the estimated network of regulation strategies, contextual problems, and addictive and suicidal behaviors. The network is interconnected. Panel B of Figure 3 presents the non-standardized estimates of the centrality measures. The most central node in terms of strength, standardized influence, and betweenness is less adaptive regulation strategies (St = 0.91; Clo = 0.02; Bet = 5; and EI = 0.92). Another notable variable is the presence of contextual problems (St = 0.82; Clo = 0.02; Bet = 6; and EI = 0.82). The results for the other variables are as follows: alcohol abuse (St = 0.63; Clo = 0.02; Bet = 1; and EI = 0.62), problematic internet use (St = 0.53; Clo = 0.02; Bet = 1; and EI = 0.53), suicidal behavior (St = 0.49; Clo = 0.02; Bet = 0; and EI = 0.29), substance addiction (St = 0.48; Clo = 0.01; Bet = 0; and EI = 0.48), and adaptive regulation strategies (St = 0.27; Clo = 0.01; Bet = 0; and EI = 0.07).

In panel C of Figure 3, the precision plot shows that the estimated network demonstrated adequate precision in the estimation. Unlike the boys’ sample, in most cases the estimates obtained through resampling (n = 1000) overlap with the original estimates, and none fall outside the confidence interval. Panel D of Figure 3 presents the stability indices of the edges in the estimated network. In this analysis, the stability of the strength, closeness, and influence of the variables does not fall below 0.70 when the sample is reduced by 50%, except for strength. These results suggest that the estimated network is partially stable. Therefore, caution should be used when generalizing the conclusions drawn.

## 4. Discussion

The main objective of this study was to analyze, using psychometric networks, the association between problem behaviors (i.e., alcohol abuse, substance addiction, problematic internet use, and suicidal behavior), the presence of problems within the family, school, or among peers (contextual problems), and the use of cognitive-emotion regulation strategies (adaptive regulation and less adaptive regulation). Additionally, the study aimed to analyze this relationship separately for boys and girls.

The descriptive data for this sample revealed that, compared to boys, girls presented more suicidal behavior, more maladaptive regulation, and more problematic internet use. These preliminary results are in accordance with the previous literature highlighting the higher proportion of affirmative responses among girls when suicidal behavior is evaluated [7,25], even though death by suicide occurs to a greater extent in boys. This gender paradox is partly attributable to the higher prevalence of affective disorders among girls [36] and the stronger tendency to express emotions among girls [51]. Also, in accordance with the existing literature, girls presented a greater amount of regulation strategies such as rumination, catastrophizing, blaming others, and self-blaming, expectantly more prone to exacerbating emotions than mitigating them. Finally, girls presented more problematic internet use than boys. These data deserve more attention. Although some studies have reported lower overall rates of problematic internet use among girls than boys [52,53], girls tend to spend more time on social networking sites. Therefore, gender differences [54] may depend on specific types of online activity (e.g., boys tend to develop more problematic behaviors related to online gaming, while in girls these relate more to social media platforms and online communication) [55,56].

To examine the relationships between the study variables, a psychometric network analysis was conducted using the entire sample, as well as the male and female samples separately. To our knowledge, this is the first study to explore these relationships through a psychometric network structure. Other researchers have applied this type of analysis to understand the relationship between depressive symptoms and substance use [57] or to assess the impact of protective and risk factors on suicidal behavior [58]. However, context-related issues, such as family, school, or peer group problems—which have been identified as risk factors for each of the problem behaviors studied [59]—have not yet been considered using this type of analysis. Network analysis provides a deeper understanding of how the selected problem behaviors, regulation strategies, and contextual issues are interrelated by examining and illustrating the connections between nodes while controlling for the effects of other nodes in the network.

In all three samples, the most central node in the network is less adaptive regulation strategies, which exhibits connections with contextual problems (especially in girls), problematic internet use, suicidal behavior (especially in boys), and—specifically in the female sample—with alcohol consumption. This finding reinforces the idea that inadequate emotion regulation is a key factor in addictive and suicidal behaviors in adolescents. According to the previous literature, less adaptive regulation strategies are associated with higher alcohol consumption [32], cannabis use [60], problematic internet use [61], and suicidal behavior [35]. In summary, our study reinforces the view that maladaptive emotional regulation strategies are a fundamental aspect underlying various behavioral issues. However, as we will discuss further, this relationship differs between boys and girls and across different types of behavioral problems.

Regarding problem behaviors, it is notable that alcohol abuse and substance addiction are positively related in all three of the networks analyzed, reinforcing the findings from the latest national survey that poly-drug use is common among adolescents [12]. This aligns with evidence suggesting that the use of one substance increases the likelihood of starting to use others [62]. In boys, the tendency towards poly-drug use appears as a phenomenon separate from other variables, showing an apparent disconnect from other behavioral problems, poor cognitive-emotional regulation, or contextual issues. This independence from other problems and the lack of connection with personal or contextual variables may be related to the social and cultural acceptance of alcohol and substance use in leisure contexts [63]. It suggests that, for boys, alcohol or substance use might be driven by risk factors not covered in this study, such as the pursuit of sensations or fun.

In contrast, for girls, alcohol and substance use is associated with other variables, and with pathways influenced by problematic internet use, contextual problems, and maladaptive regulation. Specifically, our study indicates that, for girls, substance use is linked to problematic aspects of their social context, and alcohol use is associated with problematic internet use and maladaptive or ineffective emotional regulation. In this regard, Verplaetse et al. [64] noted that alcohol may be more commonly used as a regulation strategy among women than men.

Problematic internet use was found to be related to maladaptive emotional regulation strategies in both girls and boys. Additionally, for girls, compulsive internet use was associated with contextual problems and served as a pathway to alcohol and substance use. This suggests that such behavior may have more complex implications for girls than for boys. According to the national survey, the prevalence of heavy alcohol consumption, daily tobacco use, and cannabis use is higher among adolescents who also exhibit compulsive internet use [18]. However, the meta-analysis by Lanthier-Labonté et al. [65] concludes that this relationship is not very clear, indicating a need for further investigation as well as a clearer conceptualization of problematic internet use [66]. In our study the association between problematic internet use and alcohol use was true only for girls, not for boys.

Lastly, suicidal behavior was found to have a significant positive relationship with less adaptive regulation strategies and contextual problems across all three samples. However, suicidal behavior did not exhibit a significant relationship with other problem behaviors, except for its positive association with substance addiction in the overall sample. Concerning our results, maladaptive cognitive-emotion regulation and contextual problems emerge as fundamental factors in addressing suicidal behavior among adolescents. Nevertheless, it is important to continue exploring the roles these addictive behaviors may play in suicide risk. Some researchers emphasize that internet use plays a significant role in suicidal behavior [25]. Additionally, other studies suggest that the relationship between suicidal behavior and addictive behaviors is more robust when examined in clinical populations, with its relevance in other populations remaining unclear [67].

A notable aspect of the analyzed networks is the greater interconnection between contextual problems, maladaptive cognitive emotion regulation strategies, addictive behaviors, and suicidal behaviors observed among girls. This interconnection aligns with the observance of greater emotional difficulties in girls [7,36]. Interesting, although girls seem to cope with more simultaneous difficulties, as problematic internet use, alcohol use, or substance addiction, our network analyses show that girls are more efficient than boys in avoiding suicidal behavior by using their adaptive cognitive-emotion regulation strategies. Adaptive strategies such as positive reevaluation, or putting things into perspective, are considered by some authors to distinguish the best regulators [68] and are activated by girls specifically in the face of suicidal behavior. Thus, our results suggest that girls may be more efficient in dealing with suicidal behaviors than boys. In contrast, cognitive-emotion regulation and context difficulties are associated with a wider range of behavioral problems in girls than in boys. Therefore, our results suggest that, even when emotion regulation and context difficulties are involved in a wider range of addictive problems in girls than in boys, girls may deal more efficiently with suicidal behaviors than boys. This may be due to girls’ earlier maturation rate, in addition to the fact that they have to learn to manage emotions and situations in a more complex way due to a more demanding environment.

Finally, another noteworthy finding across all three networks is the repeated positive association between less adaptive regulation strategies and contextual problems. Context during adolescence, which includes adolescents’ family, school, and peers, plays a crucial role in psychosocial development [69]. Adolescents gain new experiences and learning opportunities from their context, providing them with useful tools and skills for life, including cognitive and emotional regulation strategies [70]. It is expected that if an adolescent’s context is marked by persistent problems, such as frequent conflicts with their parents, they are more likely to lack adaptive strategies for managing the challenges they may encounter during this period [71]. Similarly, contextual problems were found to be related to substance use and problematic internet use in the total sample and were consistently linked with suicidal behaviors. These results are supported by the previous literature, which identifies family conflicts, peer problems, social isolation, and school issues as risk factors for substance use [6], problematic internet use [72], and suicidal behavior [73]. As mentioned earlier, it is noteworthy that the node of contextual problems is more significant in the female sample compared to the male sample. Additionally, while this node is positively related to substance addiction, problematic internet use, and suicidal behavior in girls, it is only associated with suicidal behavior in boys. This raises the question of why contextual problems have a greater impact on problem behaviors in this demographic. This greater emphasis on context for girls may be due to the fact that they experience a greater number of more challenging psychosocial demands than boys. In addition, Van Droogenbroeck et al. [74] suggest other possible explanations, such as an underestimation of distress by boys or the influence of gender roles, which portray girls as more sensitive to stressors.

The problem behaviors addressed in this study are complex and multifactorial, requiring thorough investigation [11,66,75]. This study aims to contribute information on their management, highlighting the roles of cognitive-emotional regulation and context. However, further research is necessary, especially studies incorporating additional variables of interest and employing analyses that explore relationships in greater detail, such as psychometric networks. It is also crucial to account for gender differences when examining these phenomena. For example, studies have shown that while girls experience more related mental health issues, they are underrepresented in treatment services [76]. Additionally, a “gender paradox” applies to suicidal behavior: rates of suicide attempts are higher in women, but rates of completed suicide are higher in men [25,36,77]. These aspects need to be considered in future research.

This study is not without limitations. First, it is a cross-sectional study, which prevents the establishment of “causal” relationships in the sense of more robust statistical control and temporal dynamics. Thus, while it identifies relationships, these need further exploration as processes are dynamic and, therefore, longitudinal [49]. Second, the variables “substance addiction”, “suicidal behavior”, and “contextual problems” encompass diverse information. For instance, cannabis use differs from cocaine use, and suicidal ideation is distinct from suicide attempts. Likewise, problems at school differ from those at home. Future studies should consider using more specific variables to examine these phenomena in greater detail. Third, data were collected through self-report questionnaires. Although these instruments generally have good psychometric properties, it would have been useful to include a response inference scale to assess respondent attentiveness. Additionally, incorporating a multi-informant perspective would have been beneficial for this study. Fourth, some of the estimated networks showed only partial stability, which could limit their generalizability. Fifth, regarding psychometric network analysis, a multigroup analysis is needed to determine whether the structure and/or edges vary between boys and girls. While psychometric networks can usefully inform an understanding of the phenomenon under study, caution should be exercised when interpreting the results: as with any cross-sectional and correlational study, the effects observed may be spurious due to the presence of unmeasured variables. Finally, the wide age range of the sample in this study encompasses different developmental stages and processes (e.g., cognitive, emotional, biological, and psychosexual) as well as contextual demands, which were not taken into account in the network analysis. These variables, closely linked to chronological age, may have significant implications and could potentially moderate the findings of the present study. Future research should examine age-related differences in specific developmental processes within the framework of network analysis. Additionally, the ecological and historical context, including the socio-cultural characteristics of the Spanish setting in which the research was conducted, may have influenced the results. These factors should also be taken into account in future studies.

On the other hand, this study also has several strengths. First, it benefits from a substantial sample size, comprising 758 adolescents and young adults. Second, the use of psychometric network analysis is noteworthy. Despite its limitations [78], this approach provides valuable insights into relationships and phenomena [49]. Third, the study is innovative, addressing current issues and providing new data that contribute to the understanding of both substance-related and non-substance-related addictive behaviors, as well as suicidal behavior.

In conclusion, it is crucial to emphasize the need for a collaborative approach between science and practice. To promote psychological well-being, especially during vulnerable periods such as childhood and adolescence, it is essential to implement effective interventions based on scientific research. In this regard, addressing maladaptive regulation strategies and contextual problems appears to be central to improving adolescents’ psychological well-being, which will likely lead to the better management of the problematic behaviors discussed.

## 5. Conclusions

The results of this study highlight that the use of ineffective or maladaptive emotional regulation strategies and the presence of problems in the developmental context are crucial aspects in the prevention of suicidal behaviors in adolescents. The study also warns of relevant gender differences. Specifically, suicidal behaviors could be better managed by girls due to the activation of effective emotional regulation strategies such as putting things into perspective, focusing on the positive, acceptance, and positive reevaluation. This differential effect contributes to understanding why boys commit suicide at a higher rate than girls since it is less likely that they can inhibit the associated negative emotions once the behaviors of suicidal ideation, planning, and attempt have begun. On the other hand, girls and boys may have different motivations for substance and alcohol use. Specifically, in girls these behaviors are related to ineffective emotional regulation and the presence of contextual problems, while in boys these behaviors seem to have independent motivations, and, hypothetically, could be more related to leisure and sensation seeking. Only problematic internet use is related to ineffective emotional regulation in both boys and girls. Consequently, the data obtained are consistent with the gender paradox, presenting a situation of greater vulnerability to contextual problems and addictive behaviors in girls, while suggesting a greater defenselessness in the face of suicidal behaviors in boys.

## Figures and Tables

**Table 1:** Descriptive statistics of the variables and significant differences by gender.

Variables	Sample	Mean	SD	Minimum	Maximum
Alcohol abuse	Total sample	0.52	0.95	0	4
	Boys	0.44	0.86	0	4
	Girls	0.59	1.01	0	4
Substance addiction	Total sample	0.32	0.73	0	4
	Boys	0.32	0.74	0	4
	Girls	0.32	0.71	0	4
Problematic internet * use	Total sample	2.16	1.32	0	4
	Boys	2.04	1.27	0	4
	Girls	2.26	1.35	0	4
Suicidal behavior **	Total sample	1.22	0.55	1	5
	Boys	1.14	0.46	1	5
	Girls	1.29	0.61	1	4.60
Contextual problems	Total sample	5.99	1.00	0.93	15
	Boys	5.95	1.14	0.93	15
	Girls	6.03	0.87	4.05	10.13
Adaptive regulation	Total sample	28.89	8.02	2	50
	Boys	28.15	8.28	10	50
	Girls	28.68	7.80	2	49
Less adaptive regulation **	Total sample	17.86	6.04	1	40
	Boys	16.20	5.64	1	40
	Girls	19.19	6.02	3	34

Note. *SD* = standard deviation. U-test significant difference * *p* < 0.05 and ** *p* < 0.01.

**Table 2:** Bivariate Correlations in the total sample (below the diagonal), in the boys’ sample (above the diagonal, outside the brackets), and in the girls’ sample (above the diagonal, in brackets).

Variables	1	2	3	4	5	6	7
1. Alcohol abuse	1	0.45 ** (0.4 **)	0.12 * (0.22 **)	0.16 ** (0.16 **)	0.00 (0.13 **)	0.02 (0.00)	0.12 * (0.22 **)
2. Substance addiction	0.43 **	1	0.18 ** (0.15 **)	0.23 ** (0.19 **)	0.13 * (0.21 **)	−0.02 (−0.05)	0.19 ** (0.22 **)
3. Problematic internet use	0.18 **	0.16 **	1	0.12 * (0.11 *)	0.14 * (0.32 **)	−0.02 (−0.06)	0.25 ** (0.26 **)
4. Suicidal behavior	0.17 **	0.20 **	0.12 **	1	0.42 ** (0.29 **)	0.00 (−0.07)	0.40 ** (0.32 **)
5. Contextual problems	0.07 *	0.17 **	0.23 **	0.34 **	1	−0.00 (−0.03)	0.35 ** (0.42 **)
6. Adaptive regulation	0.01	−0.04	−0.04	−0.05	−0.02	1	0.34 ** (0.11 **)
7. Less adaptive regulation	0.19 **	0.20 **	0.27 **	0.37 **	0.38 **	0.20 **	1

Note. * *p* < 0.05 and ** *p* < 0.01.

## Data Availability

The data will be available in the following repository: https://e-spacio.uned.es/home (accessed on 10 November 2024).
